# Abnormal functional connectivity associated with emotional dysregulation in children with attention-deficit/hyperactivity disorder

**DOI:** 10.3389/fpsyt.2025.1700693

**Published:** 2025-11-27

**Authors:** Sixun Li, Tingting Luo, Meiwen Wang, Mingjing Situ, Pei Liu, Yi Huang

**Affiliations:** 1 Child Mental Health Center, West China Hospital, Sichuan University, Chengdu, Sichuan, China; 2 Mental Health Center, West China Hospital, Sichuan University, Chengdu, Sichuan, China; 3 Laboratory of Child and Adolescent Psychiatry, Mental Health Center and Psychiatric Laboratory, West China Hospital, Sichuan University, Chengdu, Sichuan, China

**Keywords:** attention-deficit/hyperactivity disorder, emotional dysregulation, functional connectivity, dorsolateral prefrontal cortex, amygdala

## Abstract

**Objective:**

Emotional dysregulation(ED) is very common in children with Attention-Deficit/Hyperactivity Disorder (ADHD), ADHD children with ED experience more severe academic, social and quality-of-life dysfunction. Understanding of the neurobiological mechanisms and processes underpinning the ADHD with ED is sparse, this study aims to explore the distinct functional connectivity in specific group of ADHD children with ED by comparing with ADHD children without ED as well as typical development controls (TDCs).

**Method:**

A total of 77 ADHD with ED, 53 ADHD without ED and 55 matched typical TDCs were recruited. All participants completed clinical assessments and underwent resting-state functional MRI. Seed-based analysis was used to define the dorsolateral prefrontal cortex (DLPFC) and the amygdala, and resting-state functional connectivity (RSFC) analysis was then performed using DPABI software. We employed one-way analysis of covariance (ANCOVA) and *post-hoc* tests to identify the main RSFC differences among these three groups. Pearson correlation was used to analyze the relationship between RSFC and ED scores in the ADHD with ED group.

**Results:**

ADHD Children with ED exhibited increased RSFC between the DLPFC and the orbitofrontal middle gyrus (OFC)/precuneus, alongside decreased RSFC between the DLPFC and the triangular part of the inferior frontal gyrus (IFG), as well as between the amygdala and the left middle occipital gyrus/superior occipital gyrus/postcentral gyrus (GRF correction, voxel level p < 0.001, cluster level p < 0.05, two-tailed). Additionally, RSFC between the DLPFC and OFC was found to be negatively correlated with ED symptom severity in the ADHD with ED group (r = -0.32, p = 0.005), while no significant association was found between ED symptoms and amygdala-seeded RSFC in this group.

**Conclusion:**

We demonstrated abnormal functional connectivity between the DLPFC and the OFC, and between the amygdala and the left middle occipital gyrus/superior occipital. These findings may provide theoretical support for early identification of ADHD children with ED and intervention targets for future neuromodulation.

## 1 Background

Attention-deficit/hyperactivity disorder (ADHD) is a common neurodevelopmental disorder characterized by persistent patterns of inattention, hyperactivity, and impulsivity (1), affecting approximately 5-7% of children and 2-5% of adults globally (2). Beyond the core symptoms, emotional dysregulation (ED) has been increasingly recognized as a common and distinct feature in individuals with ADHD which estimated to affect 25–45% of children with ADHD (3). Moreover, ED is considered a major manifestation of ADHD in adults (4, 5). Individuals with ED often exhibit low frustration tolerance, temper outbursts, emotional impulsivity and mood lability (6). Furthermore, ADHD with ED had significantly more severe symptoms of ADHD (7), and more severe academic (8), social (9), and quality-of-life dysfunctioning (10). Therefore, early identification and intervention for ADHD with ED are crucial for reducing functional impairments and enhancing overall quality-of-life (6, 8, 11). Despite the clinical significance of this particular group of ADHD patients, the neurobiological mechanisms underlying the co-occurrence of ADHD and ED remain poorly understood, particularly in children, emphasizing the need for further research into the neuro-functional characteristics of this group.

Previous neuroimaging studies have implicated several key brain regions in the pathophysiology of ED in ADHD, including the prefrontal cortex (PFC) and amygdala (3). These regions are integral to emotional processing, regulation, and cognitive control (12). Among them, the dorsolateral prefrontal cortex (DLPFC) is primarily responsible for executive function and emotion regulation, the medial prefrontal cortex (mPFC) is involved in social cognitive processes, including theory of mind and understanding social emotions (12). Dysfunction in the above areas may lead to heightened emotional responses and increased impulsive behavior (13). Moreover, the amygdala is a central region for emotional responses, particularly in processing negative emotions, where abnormal connectivity with cortical areas may contribute to the pronounced ED observed in ADHD patients (14).

Altered functional connectivity among these regions may provide a neurobiological basis in ADHD patients with ED. Despite the growing recognition of ED as an important feature in a subset of ADHD patients, there is a paucity of research exploring the functional connectivity (FC) patterns specific in this particular group of ADHD patients. Until recently, only one study has subdivided ADHD patients into groups with high and low ED to explore their unique functional connectivity characteristics, and found that the effect sizes for the patterns of amygdala-cortical FC in ADHD participants with low ED were smaller than ADHD participants with the high ED and TDC groups (15).

In conclusion, few studies have systematically distinguished ADHD patients with and without ED, nor have they thoroughly examined the functional connectivity of key brain regions, such as the prefrontal cortex, in this particular group of ADHD children. Therefore, this study aims to fill this gap by conducting a seed-based resting-state functional connectivity (RSFC) analysis, focusing on the regions related to the pathophysiology of ED in ADHD, including the Dorsolateral/medial prefrontal cortex and amygdala. By comparing ADHD with ED, ADHD without ED and typically developing children (TDCs), this study seeks to identify distinct functional connectivity patterns that could clarify the neurobiological basis of ADHD with ED.

## 2 Method

### 2.1 Participants and clinical assessments

160 drug-naïve (stimulants and other psychotropic drugs) right-handed children with ADHD (aged 6–14 years) and full-scale IQ scores >80 were recruited from the Mental Health Center of West China Hospital, Sichuan University. Exclusion criteria were as follows: (a) a diagnosis or history of head trauma with loss of consciousness, (b) a history of neurological illness or other severe disease, and (c) either a diagnosis of schizophrenia, or depressive disorder, or anxiety disorder, or bipolar disorder, or pervasive developmental disorders, or intellectual disability or oppositional defiant disorder (ODD), or conduct disorder (CD). (d) IQ<80. The Chinese version of the Swanson Nolan and Pelham rating scale (SNAP-IVc) (16) was completed by parents of all participants. The clinical diagnosis of ADHD was first made by a qualified child and adolescent psychiatrist, and the research diagnostic standard was then determined by using the Chinese translated version of Kiddie Schedule for Affective Disorders and Schizophrenia for the DSM-5 (K-SADS-5) (17) according to the Diagnostic and Statistical Manual of Mental Disorders (DSM-5) criteria by two child and adolescent psychiatrists.

Behavioral and emotional assessment included the Children Behavior Checklist (CBCL) with ratings provided by parents. In this study, the operating standards of ED was determined by the total scores of the Anxiety/Depression, Attention and Aggression subscales in CBCL scale according to published research, if the T score of the sum of three subscales is ≥180, the child is considered to have ED symptoms; if the sum of T score of the three subscales is less than 180, it is considered that the child does not have ED symptom (18).

Eighty age- and sex-matched TDCs were recruited from local primary schools. In the TDC group, individuals with ADHD, ODD, conduct disorder (CD), or other Axis I psychiatric disorders were excluded using the K-SADS-PL interview. And none of them had a T-score ≥ 180 on the ED subscales. This study was approved by the Research Ethics Review Board of Sichuan University West China Hospital. Written informed consent was obtained from the parents of participants, and all of the children also gave assent to participate.

### 2.2 Image acquisition

All participants underwent the structure T1 and resting-state functional magnetic resonance imaging (rfMRI) imaging scan by using a 3-T MRI system (United Imaging). The rfMRI data were obtained with a gradient-echo echo-planar imaging sequence with the following parameters: repetition time (TR) = 1000 ms; echo time (TE) = 30 ms; flip angle = 60°; slice thickness = 2.5mm with no gap, field of view= 210 × 210mm^2^, matrix size = 84 × 84, voxel size = 2.5 × 2.5 × 2.5mm^3^. Each brain volume comprised 65 axial slices to cover the whole brain, and each functional imaging session contained 500 volumes. During scanning, participants were instructed to keep their heads still and relax with their eyes closed without falling asleep or having systematic thought. Earplugs and foam padding were used to reduce noise and head motion. 30 participants with ADHD and 25 in TDC were excluded from further analyses due to excessive head movements (>3.0 mm of translation or >3.0 degrees of rotation in any direction). At last, there were 77 ADHD children with ED, 53 ADHD children without ED and 55 TDCs.

### 2.3 Image processing

Resting state fMRI data was processed using DPABI 4.3 (19) based on MATLAB software. The first 10 volumes were removed in order to adapt to scanning noise. The remaining volumes were processed by way of the following seven steps (1): slice-timing (2); realignment of head-motion (3); spatial normalization, which was performed using the Montreal Neurological Institute (MNI) coordinate space with 2.5 × 2.5 × 2.5 mm (4); smoothing by using a 6 × 6 × 6 full-width at half maximum (FWHM) kernel (5); linear de-trending in order to reduce the influence of MRI equipment (6).; temporal band-pass filtering (0.01–0.08 Hz); and (7) the use of white matter signal, cerebrospinal fluid, and head motion scrubbing regressors as covariates. Fisher’s r-to-z transformation was utilized to improve the overall normality of the correlation prior to subsequent statistical analysis (20).

Seed-based voxel-wise FC analyses were used to evaluate the temporal correlations between each ROI’s resting-state time courses and each voxel of the whole brain. Based on the Anatomical Automatic Labeling (AAL) template, the “dorsolateral/medial prefrontal cortex (MNI coordinates: x = ± 42, y = 38, z = 20)” and “Amygdala” were defined separately as seeds to study the connectivity of other regions in the brain. Pearson’s correlation coefficient maps were generated for each participant and were converted to a z-value by Fisher’s z-transformation for subsequent statistical analysis.

### 2.4 Statistical analyses

One-way analysis of variance (ANOVA) was used to compare the age, intelligence, and SNAP-IV scores among the three groups. Chi-square test was applied to compare gender distributions. The statistical significance was set at P value less than 0.05. SPSS software version 29.0 (SPSS, Inc., Chicago, IL) were used for above statistical analyses.

To find the main RSFC differences among the three groups, a one-way analysis of covariance (ANCOVA) was conducted in a voxel-wise manner with age, intelligence, gender, SNAP-IV scores, and mean FD as nuisance covariates to increase the interpretability of the results. The corrected P value for ANCOVA was set at P < 0.05 using the Gaussian random field (GRF) method (voxel P value < 0.001, cluster P value < 0.05, two-tailed). Subsequently, the RSFC z-values with significant differences among three groups were extracted for every subject and compared between each pair of the three groups. Additionally, Pearson correlation analysis with age, IQ, and core ADHD symptoms as covariates (statistical significance level P < 0.05) was made to investigate the relationships between the RSFC values and the ED scores in ADHD+ED patients.

## 3 Results

### 3.1 Demographic features

The results showed that there were no significant differences in gender, age and intelligence among the three groups (gender: *χ^2^* = 4.57, *p* = 0.10, age: *F* = 1.31, *p* = 0.27, intelligence: *F* = 2.21, *p* = 0.09). There were significant differences in the scores of SNAP-IV attention deficit, hyperactivity/impulsivity, oppositional defiance and total scale scores among the three groups (SNAP-IV attention deficit: *F* = 73.73, *p* < 0.001, SNAP-IV hyperactivity/impulsivity: *F* = 37.17, *p* < 0.001, SNAP-IV oppositional defiance: *F* = 34.42, *p* < 0.001, SNAP-IV total score: *F* = 68.05, *p* < 0.001).The results of *post-hoc* t-test showed that: ADHD children with ED had higher SNAP-IV attention deficit, hyperactive/impulsiveness, oppositional defiance and total score than ADHD without ED and TDC group (**Table 1**).

**Table 1:** Demographic features.

	ADHD+ED (n=77)	ADHD-ED (n=53)	TDC (n=55)	*F*	*p*	Post-hoc
**Gender**						
male	63	39	36	4.57	0.10	–
female	14	14	19			
**Age(M±SD)**	8.22±1.69	8.51±1.80	8.75±2.90	1.31	0.27	–
**Intelligence(M±SD)**	107.07±12.27	111.87±15.53	112.84±11.19	2.21	0.09	–
**SNAP-IV**						
Attention	18.11±4.22	15.47±4.91	6.94±4.76	73.73	<0.001^***^	ADHD+ED>ADHD-ED>TDC
Hyperactivity	14.85±5.38	11.51±5.67	5.02±4.56	37.17	<0.001^***^	ADHD+ED>ADHD-ED>TDC
Oppositional	13.22±4.61	7.65±3.92	6.63±4.04	34.42	<0.001^***^	ADHD+ED>ADHD-ED,TDC
Total	46.18±10.88	34.63±11.10	18.58±11.77	68.05	<0.001^***^	ADHD+ED>ADHD-ED>TDC

ADHD+ED: Attention deficit hyperactivity disorder with emotion dysregulation group; ADHD-ED: Attention deficit hyperactivity disorder without emotion dysregulation group; TDC: healthy control group; M: average value; SD: Standard deviation; *p < 0.05; ***p < 0.001.

### 3.2 “Dorsolateral/medial prefrontal cortex” seed-based functional connectivity

**Figure 1:**
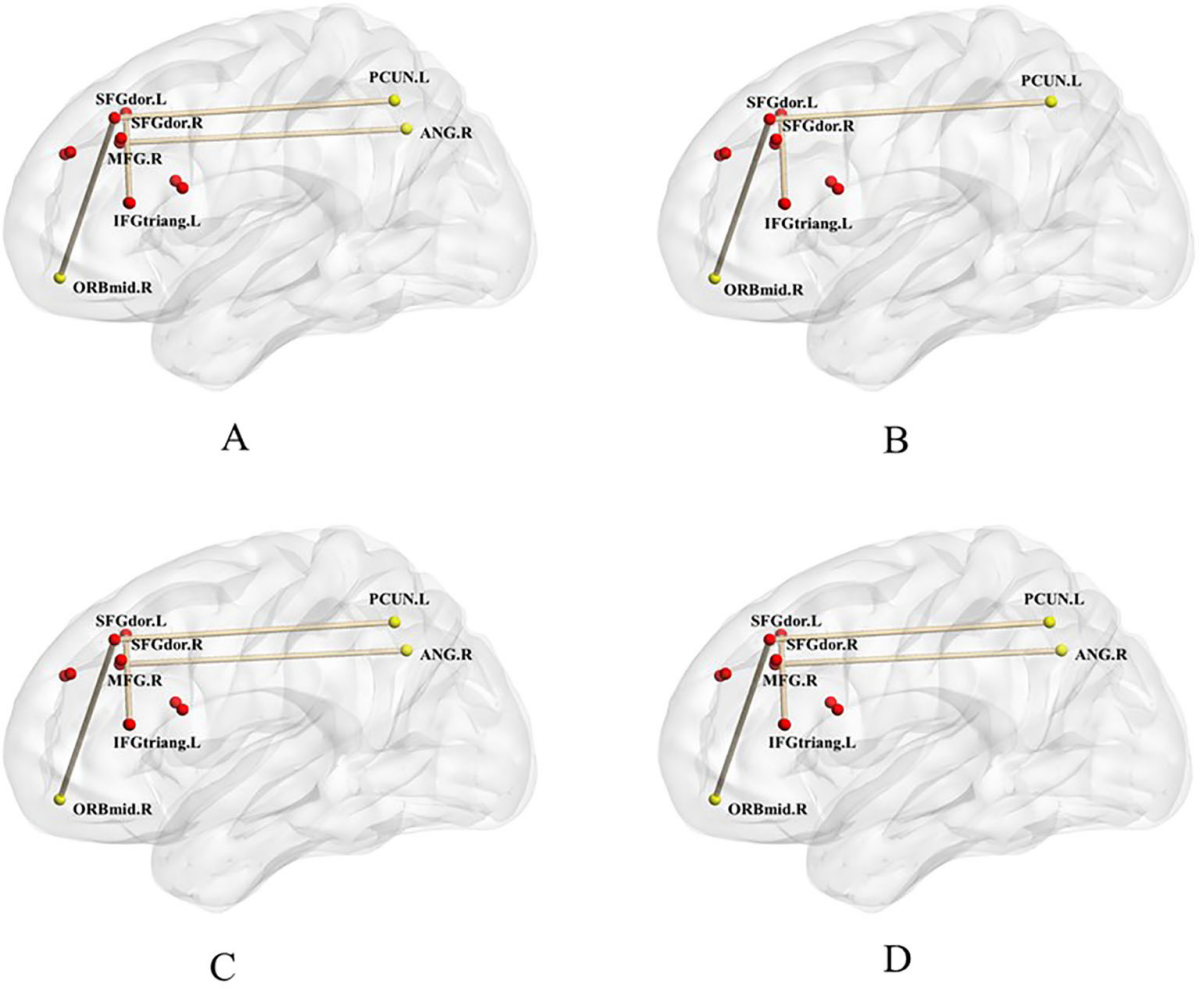
"dorsolateral/ventromedial prefrontal cortex" seed-based functional connectivity differences between the ADHD+ED, ADHD-ED, and TDC groups **(A)**, between the ADHD+ED and ADHD-ED groups **(B)**, between the ADHD+ED and TDC groups **(C)**, and between the ADHD-ED and TDC groups **(D)**. **(A)** was analyzed using ANOVA, while **(B–D)** utilized post hoc t-tests. Red dots represent the seed regions, and yellow dots indicate the brain regions showing connectivity with the seed. p < 0.05, GRF-corrected.

The ANCOVA test among the three groups revealed significant differences in RSFC values between the dorsolateral prefrontal cortex(DLPFC) and the middle orbital gyrus, precuneus and triangular inferior frontal gyrus, and between the medial prefrontal cortex and the angular gyrus (GRF correction, voxel-level p < 0.001, group-level p < 0.05, two-tailed) (**Figure 1A**; **Table 2**). *Post hoc* analysis using two-sample t-tests showed that the ADHD+ED group had significantly increased RSFC values between the DLPFC and the middle orbital gyrus (p = 0.007) and the precuneus (p = 0.008), had decreased RSFC values between the DLPFC and the triangular inferior frontal gyrus (p = 0.04) compared to the ADHD-ED and TDCs groups (**Figures 1B, C**; **Table 2**). The ADHD-ED group had increased RSFC values between the DLPFC and the middle orbital gyrus (p = 0.007) and the precuneus (p = 0.008), had decreased RSFC values between the DLPFC and the triangular inferior frontal gyrus, and between the medial prefrontal cortex and the angular gyrus compared to the TDCs group (p = 0.04) (**Figure 1D**; **Table 2**).

**Table 2:** "dorsolateral/medial prefrontal cortex" seed-based functional connectivity differences between the ADHD+ED, ADHD-ED, and TDC groups.

ROI-voxel FC	voxel	Peak MNI			Max F value	ηp²	Post-hoc
		X	Y	Z			
**SFGdor.L—ORBmid.R**	14	2.5	57.5	-12.5	12.73	0.25	ADHD-ED< ADHD+ED<TDC
SFGdor.L—PCUN.L	26	-2.5	-65	27.5	12.43	0.25	ADHD-ED< ADHD+ED<TDC
SFGdor.R—IFGtriang.L	16	-35	20	25	13.34	0.29	ADHD-ED> ADHD+ED>TDC
MFG.R—ANG.R	11	45	-52.5	37.5	15.71	0.28	ADHD-ED, ADHD+ED<TDC

MNI, Montreal Neurological Institute; x, y, z refer to the coordinates of primary peak locations in MNI space; ADHD+ED: Attention deficit hyperactivity disorder with emotion dysregulation group; ADHD-ED: Attention deficit hyperactivity disorder without emotion dysregulation group; p < 0.05, GRF-corrected. Age, sex, IQ, mean framewise displacement (FD), and ADHD core symptoms were regressed as covariates.

### 3.3 “Amygdala” seed-based functional connectivity

**Figure 2:**
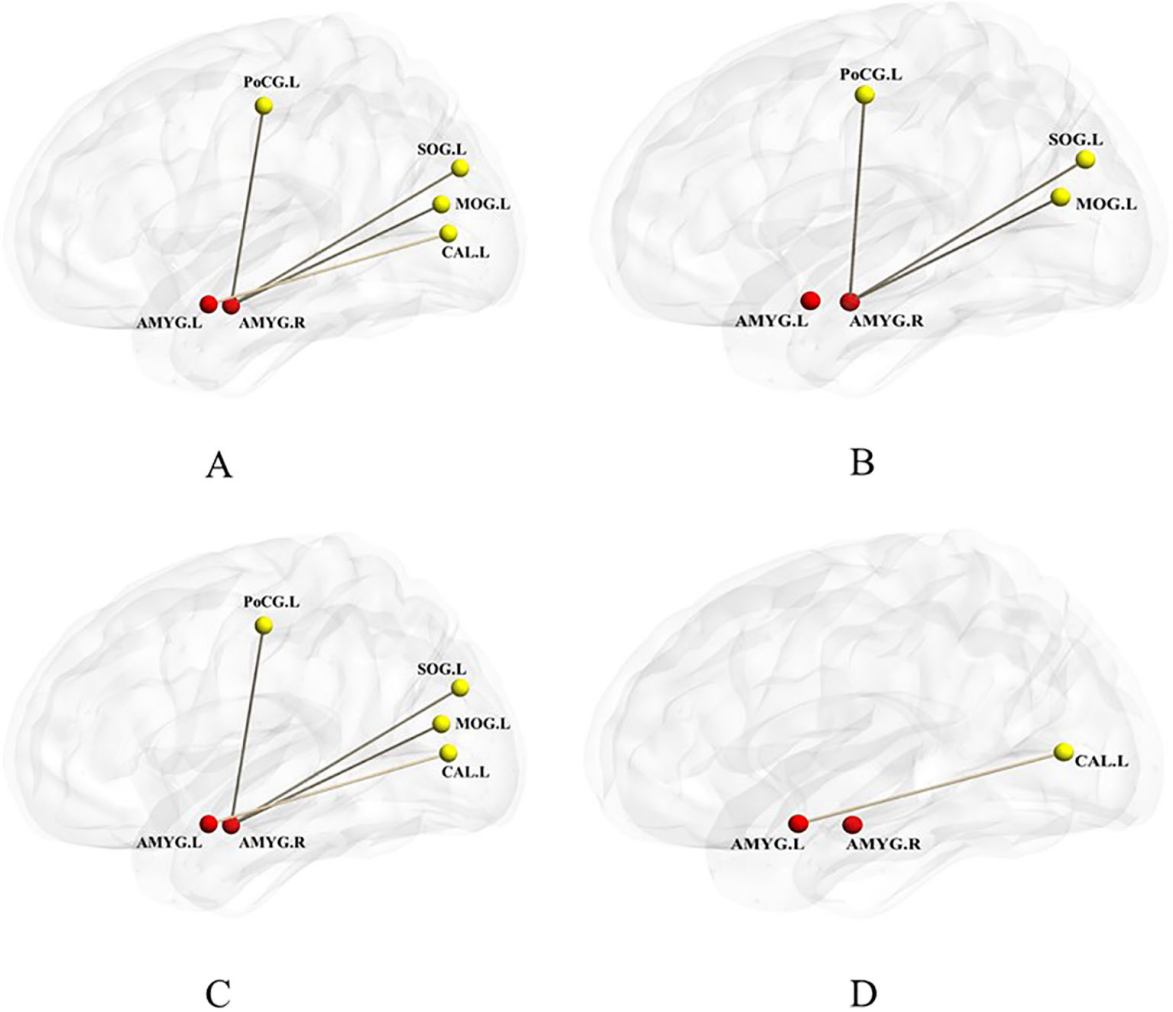
"amygdala" seed-based functional connectivity differences between the ADHD+ED, ADHD-ED, and TDC groups **(A)**, between the ADHD+ED and ADHD-ED groups **(B)**, between the ADHD+ED and TDC groups **(C)**, and between the ADHD-ED and TDC groups **(D)**. **(A)** was analyzed using ANOVA, while **(B–D)** utilized post hoc t-tests. Red dots represent the seed regions, and yellow dots indicate the brain regions showing connectivity with the seed. p < 0.05, GRF-corrected.

The ANCOVA test among the three groups revealed significant differences in RSFC values between the left amygdala and the left cuneus, as well as between the right amygdala and the left middle occipital gyrus, left superior occipital gyrus and left postcentral gyrus (GRF correction, voxel-level p < 0.001, cluster-level p < 0.05, two-tailed) (**Figure 2A**; **Table 3**). *Post hoc* analysis using two-sample t-tests showed that the ADHD+ED group exhibited decreased RSFC values between the right amygdala and the left middle occipital gyrus (p < 0.001), between the right amygdala and the left superior occipital gyrus (p = 0.04), between the right amygdala and the left postcentral gyrus (p = 0.003) compared to the ADHD-ED group (**Figure 2B**; **Table 3**). In comparison to the typically developing controls (TDCs), the ADHD-ED group also exhibited decreased RSFC values between the left amygdala and the left cuneus (p = 0.007**) (**Figures 2C, D**; **Table 3**). The ADHD+ED group showed decreased RSFC values between the left amygdala and the left cuneus (p < 0.001), and between the right amygdala and the left middle occipital gyrus (p = 0.003), between the right amygdala and the left superior occipital gyrus (p = 0.009), between the right amygdala and the left postcentral gyrus (p = 0.04).

**Table 3:** "Amygdala" seed-based functional connectivity differences between the ADHD+ED, ADHD-ED, and TDC groups.

ROI-voxel FC	voxel	Peak MNI			Max F value	ηp²	Post-hoc
		X	Y	Z			
Amy.L—CUN.L	32	-15	-67.5	22.5	7.08	0.21	ADHD+ED, ADHD-ED<TDC
Amy.R—MOG.L	23	-27.5	-77.5	15	7.85	0.14	ADHD+ED< ADHD-ED,TDC
Amy.R—SOG.L	43	-20	-65	20	3.84	0.18	ADHD+ED< ADHD-ED,TDC
Amy.R—PoCG.L	23	-37.5	-40	60	4.58	0.12	ADHD+ED< ADHD-ED,TDC

MNI, Montreal Neurological Institute; x, y, z refer to the coordinates of primary peak locations in MNI coordinates; ADHD+ED: Attention deficit hyperactivity disorder with emotion dysregulation group; ADHD-ED: Attention deficit hyperactivity disorder without emotion dysregulation group; p < 0.05, GRF-corrected. Age, IQ and ADHD core symptoms were regressed as covariates.

### 3.4 Correlations between the functional connections and emotion dysregulation severity

**Figure 3:**
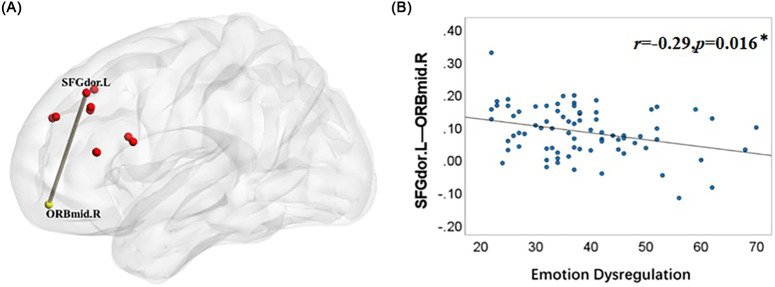
Correlation between functional connectivity and ED severity in ADHD+ED group. **(A)** The functional connectivity that correlates with ED severity in the ADHD+ED group is shown. **(B)** A negative correlation was observed between the functional connectivity values between the dorsolateral superior frontal gyrus and the orbitofrontal cortex and ED severity, **p < 0.01, Bonferroni-corrected. ED severity is assessed by the total scores of the Anxiety/Depression, Attention, and Aggression subscales on the CBCL scale.

To further explore the relationship between “dorsolateral/medial prefrontal cortex” and “amygdala” seed-based functional connectivity and ED severity in ADHD children with ED, we conducted a Pearson correlation analysis. The results revealed that the RSFC between the “DLPFC” and the “middle orbital gyrus” was negatively correlated with ED scores in the ADHD+ED group (r = -0.29, p = 0.016), while no significant association was observed between amygdala-seeded RSFC and ED symptoms scores in the ADHD+ED group (p > 0.05) (**Figure 3**).

## 4 Discussion

This study highlights the distinct functional connectivity patterns in ADHD children with ED. The findings revealed that ADHD children with ED exhibit abnormalities in functional connectivity involving both the prefrontal cortex and the amygdala. The study further explores the functional connectivity related to ED in ADHD children with ED by comparison with ADHD children without ED and TD, revealing that ED in ADHD children is primarily associated with abnormal functional connectivity between the prefrontal cortex and the orbitofrontal cortex. Overall, this study sheds light on the neurobiological underpinnings of ED in children with ADHD.

Our results indicated that the ADHD children with ED had increased resting-state functional connectivity (RSFC) between the dorsolateral prefrontal cortex (DLPFC) and the orbitofrontal cortex (OFC)/precuneus, and decreased RSFC between the DLPFC and the triangular inferior frontal gyrus(IFG), compared to the ADHD patients without ED. Several lines of evidence suggest that a fronto-striato-thalamic circuit has been involved in etiology of ADHD, comprising reciprocal connections between the caudate, putamen, thalamus, supplementary motor area, lateral prefrontal cortex, and parietal lobe. This circuit is critical to executive functions, including working memory and inhibitory control, known to be impaired in ADHD (21, 22). Additionally, a second fronto-striatal circuit involving the nucleus accumbens and orbitofrontal cortex has been proposed to be associated with ADHD (23–25). Dysfunction within this loop may underlie deficits in delay of gratification, reinforcement sensitivity, and effort-related decision making, which are characteristic of ADHD motivation and emotional regulation styles (26). Moreover, previous studies in youth with major depressive disorder(MDD) and/or generalized anxiety disorder (GAD), the common two comorbidity with ADHD, consistently implicate abnormally increased RSFC in PFC areas including the pregenual ACC and subgenual ACC, and dorsomedial and ventromedial divisions of the PFC (27), and reduced functional connectivity between the precuneus and the dorsolateral prefrontal cortex (dlPFC) (28, 29). Together with our results in ADHD with ED, it can be inferred that functional connectivity between the DLPFC and the orbitofrontal cortex (OFC)/precuneus as well as the triangular inferior frontal gyrus(IFG) maybe involved in the ED in ADHD. And abnormalities in these functional connections may serve as biological markers for the cross-disease diagnosis of ED.

We further found that “dorsolateral frontal gyrus - orbital frontal “functional connectivity was negatively correlated with ED scores in ADHD children, suggesting that functional connectivity between dorsolateral frontal gyrus and orbital frontal may serve as an indicator of the severity of emotional dysregulation in ADHD. This is consistent with previous studies suggesting that the neuroimaging mechanisms of ED in ADHD patients may involve both the dorsolateral prefrontal cortex (DLPFC) and the orbitofrontal cortex (OFC), and the OFC is implicated in deficits related to the early orienting and perception of emotional stimuli (3). The inverse relationship—where diminished connectivity is associated with more severe symptoms—suggests that this alteration is maladaptive rather than compensatory. It is plausible that ineffective integration between the cognitive control functions of the DLPFC and the reward/valence processing of the OFC underpins the poor emotional self-regulation observed in these patients. Specifically, the DLPFC, which bridges cognition and emotion, may contribute to the abnormal allocation of attention to emotional stimuli in ADHD children with ED. This regions perhaps provide a clue for the future targeted neuromodulation (e.g., neurofeedback, transcranial magnetic stimulation) of ED in ADHD.

In this study, amygdala seed-based functional connectivity analysis indicated that ADHD with ED had decreased FC between the amygdala and the left middle occipital gyrus/superior occipital gyrus/postcentral gyrus compared to ADHD without ED group, This is consistent with previous studies on irritability in youth with disruptive behavior and mood disorders, in which middle occipital gyrus was also involved (30). However, we did not replicate the study on high ED group, and revealing an enhanced intrinsic functional connectivity patterns between the amygdala–ACC/insula/STG in the high ED group (15).The main reasons for our study not replicating these findings are as follows, firstly, earlier studies quantified ED symptoms using specific subscale items from the Conners’ Parent Rating Scale, which are closely tied to comorbided anxiety and depression symptoms in ADHD. In contrast, our study employed the total scores of the Anxiety/Depression, Attention, and Aggression subscales from the CBCL scale to measure ED severity, offering a more comprehensive and independent assessment. Second, our research specifically focused on the unique ADHD with ED subtype, minimizing the impact of potential confounders. Thirdly, there may exit an abnormal pattern of functional connectivity between the amygdala and the ACC, due to the limited sample size after grouping, we were unable to detect the above abnormalities. Future research needs to further validate this result using standardized assessment tools with larger sample sizes.

Several limitations should be acknowledged in our study. Firstly, this study selected the dorsolateral/ventromedial prefrontal and amygdala as seed points for functional connectivity analysis. However, previous studies suggest that multiple brain regions (such as ACC) are also involved in ADHD+ED. In future research, we will further increase studies in other related regions. Secondly, we selected the total sores of the Anxiety/Depression, Attention and Aggression subscales in CBCL scale for measuring ED severity. However, it remains uncertain whether alternative tools assessing ED would yield similar results. Thirdly, this study is a cross-sectional study, which limits the reliability of interpreting and verifying the results from a longitudinal dynamic perspective. Future investigations incorporating longitudinal approaches may provide insights into these complex relationships. Lastly, it is important to note that our study utilized a relatively modest sample size. Conducting future studies with larger samples will enhance the generalizability of our findings.

## 5 Conclusion

In conclusion, this study extends previous research regarding the neurobiology of emotion dysregulation in ADHD children. We demonstrated abnormal functional connectivity between the DLPFC and the OFC, and between the amygdala and the left middle occipital gyrus/superior occipital. These findings may provide theoretical support for early identification of ADHD children with emotion dysregulation and intervention targets for future neuromodulation.

## Data Availability

The raw data supporting the conclusions of this article will be made available by the authors, without undue reservation.

## References

[1] Faraone SV Bellgrove MA Brikell I Cortese S Hartman CA Hollis C . Attention-deficit/hyperactivity disorder. Nat Rev Dis Primers. (2024) 10(1):29. doi: 10.1038/s41572-024-00495-0, PMID: 38622144

[2] Polanczyk G de Lima MS Horta BL Biederman J Rohde LA . The worldwide prevalence of ADHD: a systematic review and metaregression analysis. Am J Psychiatry. (2007) 164:942–8. doi: 10.1176/ajp.2007.164.6.942, PMID: 17541055

[3] Shaw P Stringaris A Nigg J Leibenluft E . Emotion dysregulation in attention deficit hyperactivity disorder. Am J Psychiatry. (2014) 171:276–93. doi: 10.1176/appi.ajp.2013.13070966, PMID: 24480998 PMC4282137

[4] Hirsch O Chavanon M Riechmann E Christiansen H . Emotional dysregulation is a primary symptom in adult Attention-Deficit/Hyperactivity Disorder (ADHD). J Affect Disord. (2018) 232:41–7. doi: 10.1016/j.jad.2018.02.007, PMID: 29477097

[5] Soler-Gutiérrez A-M Pérez-González J-C Mayas J . Evidence of emotion dysregulation as a core symptom of adult ADHD: A systematic review. PloS One. (2023) 18:e0280131. doi: 10.1371/journal.pone.0280131, PMID: 36608036 PMC9821724

[6] Surman CBH Biederman J Spencer T Miller CA McDermott KM Faraone SV . Understanding deficient emotional self-regulation in adults with attention deficit hyperactivity disorder: a controlled study. Attention deficit hyperactivity Disord. (2013) 5:273–81. doi: 10.1007/s12402-012-0100-8, PMID: 23413201 PMC4009378

[7] Biederman J DiSalvo M Woodworth KY Fried R Uchida M Biederman I . Toward operationalizing deficient emotional self-regulation in newly referred adults with ADHD: A receiver operator characteristic curve analysis. Eur psychiatry: J Assoc Eur Psychiatrists. (2020) 63:e21. doi: 10.1192/j.eurpsy.2019.11, PMID: 32093797 PMC7315889

[8] Barkley RA Fischer M . The unique contribution of emotional impulsiveness to impairment in major life activities in hyperactive children as adults. J Am Acad Child Adolesc Psychiatry. (2010) 49:503–13. doi: 10.1097/00004583-201005000-00011, PMID: 20431470

[9] Lee CA Milich R Lorch EP Flory K Owens JS Lamont AE . Forming first impressions of children: the role of attention-deficit/hyperactivity disorder symptoms and emotion dysregulation. J Child Psychol psychiatry Allied disciplines. (2018) 59:556–64. doi: 10.1111/jcpp.12835, PMID: 29083026

[10] Ben-Dor Cohen M Eldar E Maeir A Nahum M . Emotional dysregulation and health related quality of life in young adults with ADHD: a cross sectional study. Health Qual Life outcomes. (2021) 19:270. doi: 10.1186/s12955-021-01904-8, PMID: 34930314 PMC8691086

[11] Skirrow C Asherson P . Emotional lability, comorbidity and impairment in adults with attention-deficit hyperactivity disorder. J Affect Disord. (2013) 147:80–6. doi: 10.1016/j.jad.2012.10.011, PMID: 23218897

[12] Blakemore S-J . The social brain in adolescence. Nat Rev Neurosci. (2008) 9:267–77. doi: 10.1038/nrn2353, PMID: 18354399

[13] Davidson RJ Putnam KM Larson CL . Dysfunction in the neural circuitry of emotion regulation–a possible prelude to violence. Sci (New York NY). (2000) 289:591–4. doi: 10.1126/science.289.5479.591, PMID: 10915615

[14] Adolphs R Gosselin F Buchanan TW Tranel D Schyns P Damasio AR . A mechanism for impaired fear recognition after amygdala damage. Nature. (2005) 433:68–72. doi: 10.1038/nature03086, PMID: 15635411

[15] Hulvershorn LA Mennes M Castellanos FX Di Martino A Milham MP Hummer TA . Abnormal amygdala functional connectivity associated with emotional lability in children with attention-deficit/hyperactivity disorder. J Am Acad Child Adolesc Psychiatry. (2014) 53:351–61.e1. doi: 10.1016/j.jaac.2013.11.012, PMID: 24565362 PMC3961844

[16] Gau SSF Shang CY Liu SK Lin CH Swanson JM Liu YC . Psychometric properties of the Chinese version of the Swanson, Nolan, and Pelham, version IV scale–parent form. Int J Methods Psychiatr Res. (2008) 17:35–44. doi: 10.1002/mpr.237, PMID: 18286459 PMC6878250

[17] Dun Y Li Q-R Yu H Bai Y Song Z Lei C . Reliability and validity of the Chinese version of the kiddie-schedule for affective disorders and schizophrenia-present and lifetime version DSM-5 (K-SADS-PL-C DSM-5). J Affect Disord. (2022) 317:72–8. doi: 10.1016/j.jad.2022.08.062, PMID: 36029880

[18] Biederman J Spencer T Lomedico A Day H Petty CR Faraone SV . Deficient emotional self-regulation and pediatric attention deficit hyperactivity disorder: a family risk analysis. Psychol Med. (2012) 42:639–46. doi: 10.1017/S0033291711001644, PMID: 21861953

[19] Yan C-G Wang X-D Zuo X-N Zang Y-F . DPABI: data processing & Analysis for (Resting-state) brain imaging. Neuroinformatics. (2016) 14:339–51. doi: 10.1007/s12021-016-9299-4, PMID: 27075850

[20] Liu Y Yu C Liang M Li J Tian L Zhou Y . Whole brain functional connectivity in the early blind. Brain: J Neurol. (2007) 130:2085–96. doi: 10.1093/brain/awm121, PMID: 17533167

[21] Norman LJ Carlisi C Lukito S Hart H Mataix-Cols D Radua J . Structural and functional brain abnormalities in attention-deficit/hyperactivity disorder and obsessive-compulsive disorder: A comparative meta-analysis. JAMA Psychiatry. (2016) 73:815–25. doi: 10.1001/jamapsychiatry.2016.0700, PMID: 27276220

[22] Arnsten AFT Rubia K . Neurobiological circuits regulating attention, cognitive control, motivation, and emotion: disruptions in neurodevelopmental psychiatric disorders. J Am Acad Child Adolesc Psychiatry. (2012) 51:356–67. doi: 10.1016/j.jaac.2012.01.008, PMID: 22449642

[23] Bralten J Greven CU Franke B Mennes M Zwiers MP Rommelse NNJ . Voxel-based morphometry analysis reveals frontal brain differences in participants with ADHD and their unaffected siblings. J Psychiatry neuroscience: Jpn. (2016) 41:272–9. doi: 10.1503/jpn.140377, PMID: 26679925 PMC4915936

[24] Albaugh MD Ivanova M Chaarani B Orr C Allgaier N Althoff RR . Ventromedial prefrontal volume in adolescence predicts hyperactive/inattentive symptoms in adulthood. Cereb Cortex (New York NY: 1991). (2019) 29:1866–74. doi: 10.1093/cercor/bhy066, PMID: 29912404 PMC6458906

[25] Albaugh MD Orr C Chaarani B Althoff RR Allgaier N D'Alberto N . Inattention and reaction time variability are linked to ventromedial prefrontal volume in adolescents. Biol Psychiatry. (2017) 82:660–8. doi: 10.1016/j.biopsych.2017.01.003, PMID: 28237458 PMC5509516

[26] Plichta MM Scheres A . Ventral-striatal responsiveness during reward anticipation in ADHD and its relation to trait impulsivity in the healthy population: a meta-analytic review of the fMRI literature. Neurosci Biobehav Rev. (2014) 38:125–34. doi: 10.1016/j.neubiorev.2013.07.012, PMID: 23928090 PMC3989497

[27] Kerestes R Davey CG Stephanou K Whittle S Harrison BJ . Functional brain imaging studies of youth depression: a systematic review. NeuroImage Clinical. (2014) 4:209–31. doi: 10.1016/j.nicl.2013.11.009, PMID: 24455472 PMC3895619

[28] Wang W Hou J Qian S Liu K Li B Li M . Aberrant regional neural fluctuations and functional connectivity in generalized anxiety disorder revealed by resting-state functional magnetic resonance imaging. Neurosci Lett. (2016) 624:78–84. doi: 10.1016/j.neulet.2016.05.005, PMID: 27163197

[29] Li W Cui H Zhu Z Kong L Guo Q Zhu Y . Aberrant functional connectivity between the amygdala and the temporal pole in drug-free generalized anxiety disorder. Front Hum Neurosci. (2016) 10:549. doi: 10.3389/fnhum.2016.00549, PMID: 27867352 PMC5095112

[30] Suk JW Blair RJR Vaughan B Lerdahl A Garvey WF Edwards R . Mediating effect of amygdala activity on response to fear vs. happiness in youth with significant levels of irritability and disruptive mood and behavior disorders. Front Behav Neurosci. (2023) 17:1204574. doi: 10.3389/fnbeh.2023.1204574, PMID: 37901308 PMC10602729

